# Short‐ Versus Long‐Term Hearing Preservation and Speech Recognition Outcomes in Precurved and Straight Electrode Arrays

**DOI:** 10.1002/oto2.70254

**Published:** 2026-05-20

**Authors:** Lily V. Den Hartog, Andrew W. Liu, Divya A. Chari

**Affiliations:** ^1^ University of Massachusetts Chan School of Medicine Worcester Massachusetts USA; ^2^ Department of Otolaryngology–Head and Neck Surgery UMass Memorial Medical Center Worcester Massachusetts USA; ^3^ Department of Otolaryngology–Head and Neck Surgery Massachusetts Eye and Ear Boston Massachusetts USA

**Keywords:** cochlear implant, electrode design, hearing preservation, precurved electrode, speech perception, straight electrode

## Abstract

**Objective:**

Compare audiologic outcomes between precurved and straight electrode arrays in cochlear implant (CI) recipients while controlling for age and preoperative hearing in a large cohort.

**Study Design:**

Retrospective chart review.

**Setting:**

Tertiary referral center.

**Methods:**

Adult CI recipients who underwent surgery from 2018 to 2025 with Cochlear Americas slim modiolar CI532/632 (precurved) or slim straight CI522/622 (straight) electrode arrays were included. Preimplantation and postimplantation low‐frequency pure tone average (LFPTA) in the short‐ (1‐ and 6‐months) and long‐term (≥12 months) and speech recognition testing (CNC, AzBio in quiet) at 1‐, 3‐, 6‐, and 12‐months postactivation were recorded. Hearing preservation (LFPTA shift) and speech recognition scores were evaluated using multivariate linear regression analysis.

**Results:**

One hundred seventy‐two patients (190 ears) were included. 124 (65.3%) and 66 (34.7%) ears were implanted with a precurved or straight electrode, respectively. 47 (24.7%) ears had residual hearing (LFPTA ≤ 80) preoperatively. Precurved electrode recipients had significantly better hearing preservation compared to straight electrode recipients in the short‐term (*F* = 4.016, *P* = .039), though this advantage diminished in long‐term follow‐up after controlling for age, sex, and preoperative LFPTA. CNC and AzBio scores were not significantly different between electrode arrays at any timepoint postimplantation.

**Conclusion:**

Precurved electrodes were associated with small but statistically significant greater hearing preservation in the short‐term compared to straight electrodes, but this difference did not persist in the long‐term. Speech recognition outcomes were similar for precurved and straight electrodes at all timepoints. These findings should be considered during preoperative decision‐making for potential CI recipients.

Cochlear implantation has revolutionized the treatment of moderate to profound sensorineural hearing loss.^1^, ^2^ Despite significant advances in device design and surgical techniques, speech recognition outcomes and hearing preservation rates remain highly variable among cochlear implant (CI) recipients,^3^, ^4^, ^5^, ^6^, ^7^, ^8^ with performance differences that can substantially impact quality of life and functional communication abilities.^9^, ^10^, ^11^, ^12^ Multiple factors contribute to this variability, including patient‐specific characteristics such as age at implantation, etiology of hearing loss, medical comorbidities, duration of hearing loss, preoperative hearing levels, and surgical approaches, and device‐related factors including electrode array design and intracochlear positioning.^3^, ^4^, ^13^, ^14^, ^15^, ^16^, ^17^, ^18^, ^19^

Electrode design has emerged as a critical determinant of postoperative outcomes, with 2 primary configurations available: precurved and straight electrode arrays.^20^ Precurved electrodes conform closely to the modiolus, theoretically providing closer proximity to spiral ganglion neurons and enabling more efficient neural stimulation with reduced current spread.^21^, ^22^, ^23^, ^24^, ^25^, ^26^, ^27^ However, the precurved electrode may confer increased risk of scalar translocation,^28^, ^29^ which has been associated with destruction of residual hearing.^3^, ^28^, ^30^, ^31^, ^32^, ^33^ In contrast, straight electrodes are designed to curve along the outer wall of the scala tympani during insertion, minimizing the risk of scalar translocation and thereby lessening possible insertion trauma to the spiral ganglion, but are obligately farther from the modiolus.^28^, ^31^, ^34^, ^35^

Several studies suggest that reduced electrode‐to‐modiolus distance is associated with improved speech perception outcomes, particularly in the long‐term.^21^, ^25^, ^36^, ^37^, ^38^, ^39^, ^40^ However, comparisons between electrode array designs have yielded mixed results, likely due to heterogeneity in study design, sample size, and device generation.^32^, ^33^, ^36^, ^37^, ^38^, ^39^, ^41^, ^42^, ^43^, ^44^, ^45^, ^46^, ^47^, ^48^, ^49^, ^50^ A stylet‐based model of precurved electrodes (Cochlear Contour Advance CI512/612) has been associated with intracochlear trauma and higher rates of scalar translocation, resulting in poorer hearing preservation rates.^28^, ^29^, ^33^, ^51^ However, the sheath‐like model of precurved slim perimodiolar electrodes (Cochlear Slim Modiolar CI532/632) is more tapered and flexible, demonstrating lower rates of translocation compared to the Cochlear 512/612 device.^52^, ^53^ Prior studies have shown that sheathed precurved electrodes may achieve superior speech performance outcomes compared to straight electrodes, but hearing preservation rates are either unchanged or slightly better with straight electrodes.^5^, ^36^, ^37^, ^38^, ^44^, ^52^

The present study aims to assess speech perception and hearing preservation outcomes among adult CI recipients implanted with either a precurved (CI532/632) or straight (CI522/622) electrode array. Drawing on a large, single‐institution cohort, we control for key demographic variables and duration of hearing loss to assess the impact of electrode design on speech perception outcomes and residual low‐frequency pure tone thresholds. These findings aim to inform clinical decision‐making amid ongoing advances in the design of precurved electrode arrays and growing surgical expertise in their insertion, particularly for patients with residual hearing who may be candidates for electroacoustic stimulation (EAS).

## Methods

### Participant Selection

Approval for this study was obtained from the University of Massachusetts Institutional Review Board (STUDY00002312). A retrospective chart review was conducted on all adult patients who underwent cochlear implantation with precurved (Cochlear Slim Modiolar Electrode, CI532/CI632; Cochlear Americas) or straight (Cochlear Slim Straight Electrode, CI522/622; Cochlear Americas) electrode arrays between January 1, 2018 and May 1, 2025 at a single academic cochlear implant center. All recipients met FDA criteria for cochlear implant candidacy. Inclusion criteria consisted of postlingually deafened adults (≥18 years) with documented preoperative speech testing and postoperative speech perception scores available at a minimum of 3 months following implantation. Patients were excluded if they underwent revision surgery or had anatomic abnormalities or cochlear malformations. In recipients of bilateral CIs, each ear was analyzed independently. Analysis of hearing preservation was limited to patients with low‐frequency (250 and 500 Hz) pure tone average (LFPTA) ≤80 dB preoperatively. Intraoperatively, the round window membrane (RWM) bony overhang was removed with a reduced drill speed of 6000 RPM and the RWM was opened using a 28‐gauge needle or Rosen needle. In select cases in which the angulation of the RWM was estimated to be greater than 45°, an extended round window approach was performed by removing additional bone around the RWM. Steroids were provided intraoperatively, both intravenously and topically near the RWM; they were not routinely provided postoperatively.

### Data Acquisition

Demographic information (age, sex, race), etiology of hearing loss, and surgical approach (pure round window, extended round window) was collected from the electronic health record. LFPTA (250 and 500 Hz) was calculated from audiograms conducted preoperatively and postoperatively, and if there was no response at the maximal output of the audiometer, the threshold was noted as 120 dB for that frequency. Functional residual hearing was determined to be preserved if the postoperative LFPTA was ≤80. LFPTA shifts were calculated by subtracting the preoperative value from the postoperative value. Postoperative LFPTA was measured at the time of device activation (~1 month postoperatively), and at 6‐ and 12‐months after activation; postoperative measurements at the time of device activation and at 6‐months after activation represented “short‐term” follow‐up and measurements at 12‐months after activation represented “long‐term” follow‐up. Consonant‐Nucleus‐Consonant Whole Word (CNC) and AzBio Sentence Test in quiet (AzBio) scores were obtained preoperatively and postoperatively at 1‐, 3‐, 6‐, and 12‐months postactivation to measure speech recognition. Speech recognition testing was completed via presentation of recorded CNC whole word lists at 60 dB SPL or recorded AzBio sentence lists in quiet at 60 dB SPL in the cochlear implant‐alone listening condition; the ear that was not being tested was either plugged or masked. Scores were recorded as the percentage of words correctly repeated.

### Data Analysis

Descriptive statistics were used to compare precurved and straight electrodes. Multivariate linear regression models with primary outcomes of CNC, AzBio, or LFPTA shift were subsequently built, including age at implantation, sex, and preoperative speech recognition testing (CNC or AzBio score) or preoperative residual hearing (LFPTA) as controlling variables. Comparison of electrode groups was conducted using the Wilcoxon rank sum test (Mann‐Whitney) for continuous variables and chi‐square test for categorical variables. All statistical analyses were performed using RStudio (R version 4.3.1; Posit PBC). A *P* < .05 was considered statistically significant.

## Results

### Speech Recognition

190 implanted ears from 172 individual patients were included for final analysis of speech recognition outcomes after implantation. Within our cohort, 124 (65.3%) and 66 (34.7%) ears were implanted with a precurved and straight electrodes, respectively. Surgical approach was predominantly round window (precurved: n = 25, straight: n = 55) or extended round window (precurved: n = 98, straight: n = 8), with a limited number of insertions performed via cochleostomy approach (precurved: n = 1, straight: n = 1) or partial drill out of the basal turn of the cochlea (precurved: n = 0, straight: n = 2).

Table 1 demonstrates demographic characteristics stratified by electrode type. The median age at implantation was 67 years (IQR 53.25‐78), 53.16% of implant recipients were male, 88.42% were white, and 52% were right‐sided. The most common etiology of hearing loss was progressive sensorineural hearing loss, affecting 61% of implant recipients, with the remainder including sudden sensorineural hearing loss (17%), Meniere's disease (8%), mixed sensorineural and conductive hearing loss (7%), infection‐related causes (5%), and congenital hearing loss (2%). Age, sex, and race were not significantly different between the 2 electrode groups. For preoperative audiologic testing, LFPTA was not significantly different between the 2 groups (*P* = .10), though speech recognition scores were significantly higher for ears implanted with a straight than with a precurved electrode: preoperative CNC scores were 11% in the straight group and 7% in the precurved group (*P* = .021), and preoperative AzBio scores were 20% in the straight group and 13% in the precurved group (*P* = .024).

**Table 1:** Characteristics of Cochlear Implantation Patients Stratified by Electrode Type

Characteristic	Straight (n = 66, 34.7%)	Precurved (n = 124, 65.3%)	*P*‐value^a^
Age at implantation^b^	67.5 (55.25‐76.75)	67 (52.75‐78.25)	.89
Sex^c^			1
Female	31 (47.0%)	58 (46.8%)	
Male	35 (53.0%)	66 (53.2%)	
Race^c^			.59
White	60 (90.9%)	108 (87.1%)	
Non‐White	6 (9.1%)	16 (12.9%)	
Preoperative LFPTA^d^	66.96 ± 24.04	72.98 ± 22.3	.10
Preoperative speech recognition			
AzBio quiet^d^	20% ± 22%	13% ± 18%	**.024**
CNC whole word^d^	11% ± 12%	7% ± 10%	**.021**

Demographic and baseline audiometric data were collected for CI patients receiving either a straight or precurved electrode. Significant *P* values are in bold.

^a^ *t*‐test (continuous) or chi‐square (categorical).

^b^ Median (interquartile range).

^c^ n (%).

^d^ Mean ± standard deviation.

**Figure 1:**
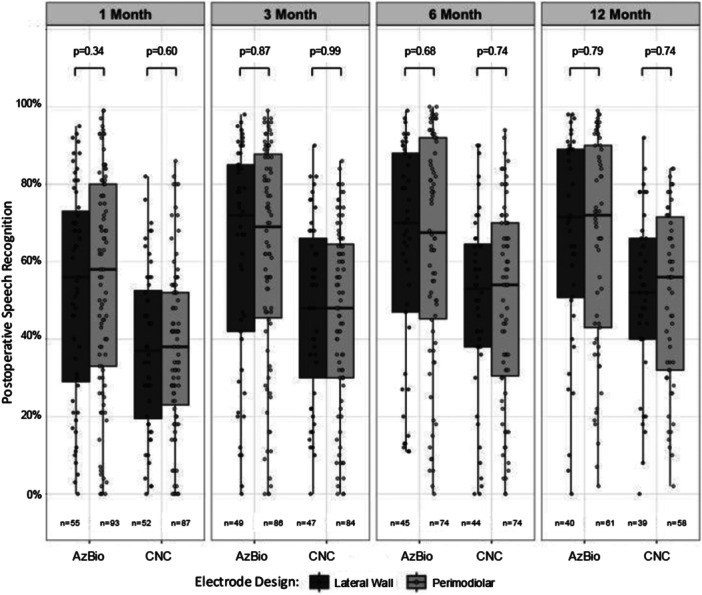
Postoperative AzBio and CNC scores stratified by electrode type. Boxplots depict AzBio and CNC scores at 4 postoperative timepoints following device activation (1‐, 3‐, 6‐, and 12‐months).

Postoperative speech recognition scores did not significantly differ between the 2 electrode array types at any timepoint, even after adjusting for potential confounding variables using multivariate linear regression (CNC: *F* = 4.21, *P* = .60 at 1‐month; *F* = 2.29, *P* = .99 at 3‐months; *F* = 1.70, *P* = .74 at 6‐months, *F* = 0.90, *P* = .74 at 12‐months; AzBio: *F* = 6.02, *P* = .34 at 1‐month; *F* = 1.86, *P* = .87 at 3‐months; *F* = 2.20, *P* = .68 at 6 months, *F* = 1.69, *P* = .79 at 12‐months; Figure 1).

Age at implantation was significantly associated with postoperative AzBio at 1‐, 3‐, and 6‐months post‐activation (*P* = .01 for all time points) and postoperative CNC (*P* < .01 at 1‐months; *P* < .05 at 3‐ and 6‐months), but age was not significantly associated with speech perception outcomes for testing performed at 12‐months for either AzBio or CNC (Table 2). Sex did not correlate with postoperative speech recognition. Preoperative speech recognition measurements were associated with their respective postoperative outcomes at some, but not all, timepoints (CNC: *P* < .05 at 1‐ and 3‐months; AzBio: *P* < .05 at 1‐ and 12‐months).

**Table 2:** Multivariate Linear Regression With Primary Outcome of CNC Whole Word Scores and AzBio Quiet Scores at 12‐Months Following Device Activation

	AzBio (n = 101)			CNC (n = 97)		
Characteristic	OR	95% confidence interval	*P*‐value	OR	95% confidence interval	*P*‐value
Age at implantation	−0.00	−0.01, 0.00	.24	−0.00	0.00, 0.0	.48
Sex	−0.07	−0.18, 0.04	.19	−0.03	−0.12, 0.06	.50
Preoperative speech recognition	0.28	0.01, 0.56	**.04**	0.36	−0.05, 0.76	.08
Electrode type	0.02	−0.10, 0.13	.79	0.02	−0.08, 0.11	.74

In the AzBio analysis, 40 ears implanted with a straight electrode and 61 ears implanted with a precurved electrode were included. In the CNC analysis, 39 ears implanted with a straight electrode and 58 ears implanted with a precurved electrode were included. Significant *P* values are in bold.

Abbreviation: OR, odds ratio.

### Hearing Preservation

47 ears were included for final analysis of hearing preservation outcomes after CI. Table 3 demonstrates demographic characteristics stratified by electrode type in the hearing preservation analysis cohort, of which 23 ears (49%) were implanted with a precurved electrode and 24 ears (51%) were implanted with a straight electrode. None of the baseline characteristics (age, sex, race) were significantly different between electrode groups. Average preoperative LFPTA was similar in the 2 groups (precurved: 50.22 ± 13.75 vs straight: 45.95 ± 13.53; *P* = .20).

**Table 3:** Characteristics of Patients With Preoperative Residual Hearing Stratified by Electrode Type

Characteristic	Straight (n = 24, 51%)	Precurved (n = 23, 49%)	*P*‐value^a^
Age at implantation^b^	67.5 (61.75‐81)	70 (59‐77)	.83
Sex^c^			.09
Female	12 (50.0%)	5 (21.7%)	
Male	12 (50.0%)	18 (78.3%)	
Race^c^			.97
White	23 (95.8%)	21 (91.3%)	
Non‐White	1 (4.2%)	2 (8.7%)	
Preoperative LFPTA^d^	45.95 ± 13.53	50.22 ± 13.75	.20

Demographic and baseline audiometric data were collected for CI recipients with residual hearing who underwent implantation with either a straight or precurved electrode.

^a^ *t*‐Test (continuous) or *x* ^2^ (categorical).

^b^ Median (interquartile range).

^c^ n (%).

^d^ Mean ± standard deviation.

While the percentage of ears retaining LFPTA < 80 was greater in the precurved electrode group compared to the straight electrode group at all postoperative timepoints, this trend did not reach statistical significance: 1‐month (68.18% vs 50.00%), 6‐month (81.82% vs 60.00%), and 12‐month (85.71% vs 75.00%).

**Figure 2:**
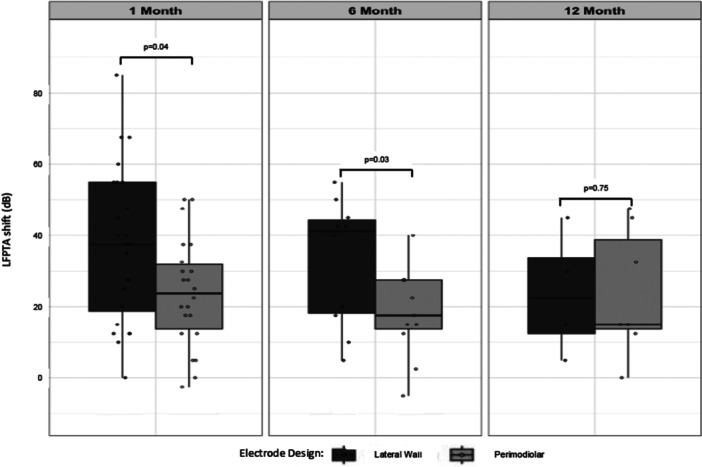
LFPTA shift stratified by electrode type. Boxplots demonstrate LFPTA shift at 3 postoperative timepoints following device activation (1‐, 6‐, and 12‐months). A smaller LFPTA shift indicates superior hearing preservation. LFPTA, low‐frequency pure tone average.

Ears implanted with a precurved electrode demonstrated a significantly lower average LFPTA shift (24.57 ± 16.49 dB) than did ears implanted with a straight electrode (38.44 ± 22.13 dB) in aggregate (*F* = 4.71, *P* = .03), indicating superior preservation of residual hearing. Subcohort analysis at each postoperative timepoint revealed that recipients of precurved electrodes had a significantly lower LFPTA shift at 1‐ and 6‐months (*F* = 4.02, *P* = .04 at 1‐month; *F* = 3.78, *P* = .03 at 6‐months), but not in long‐term follow‐up (*F* = 1.19, *P* = .75) at >12‐months (Figure 2). Multivariate regression analysis of the hearing preservation group in aggregate (Table 4) likewise demonstrated a significant effect of electrode type on LFPTA shift (OR = −12.91; 95% confidence interval: −24.29 to −1.53, *P* = .03). Age at implantation (OR = 0.65, 95% confidence interval = 0.19‐1.11, *P* < .01) was also significantly associated with LFPTA shift, while sex and preoperative LFPTA were not.

**Table 4:** Multivariate Linear Regression With Primary Outcome of LFPTA Shift

	LFPTA shift (n = 47)		
Characteristic	OR	95% confidence interval	*P*‐value
Age at implantation	0.65	0.19, 1.11	**<.01**
Sex	−2.22	−15.26, 10.82	.733
Preoperative LFPTA	−0.17	−0.59, 0.24	.407
Electrode type	−12.91	−24.29, −1.53	**.027**

24 ears implanted with a straight electrode and 23 ears implanted with a precurved electrode were included. Significant *P* values are in bold.

Abbreviations: LFPTA, low‐frequency pure tone average; OR, odds ratio.

## Discussion

In this large retrospective cohort study comparing audiologic outcomes after cochlear implantation with precurved or straight electrode arrays from a single manufacturer, perimodiolar electrodes demonstrated significantly better low‐frequency hearing preservation in the short‐term (<6‐months) and performed similarly to straight electrodes in the long‐term (>12‐months), even after controlling for patient age and preoperative low‐frequency hearing. While improved speech recognition at all postoperative timepoints demonstrated overall CI efficacy, there was no significant difference in speech perception outcomes based on electrode design in our cohort, despite significantly superior preoperative CNC and AzBio scores in the straight group compared to the precurved group.

### Hearing Preservation

As CI criteria continue to broaden, a growing population of adult CI candidates present with significant residual acoustic hearing, particularly in the low frequencies. Preserving these functional low‐frequency thresholds may enable patients to utilize combined electroacoustic stimulation, which has been shown to enhance speech understanding in challenging listening environments and address the inherent spectral resolution constraints of electrical stimulation alone.^10^, ^54^, ^55^, ^56^ Consequently, hearing preservation has become a primary surgical objective in patients who retain usable low‐frequency hearing despite inadequate benefit from traditional hearing aids.

Straight electrode design has traditionally been relied upon for hearing preservation due to reported rates of reduced cochlear trauma (eg, scalar translocation).^28^, ^51^ Indeed, a 2018 cross‐sectional survey of American Neurotology Society members reported that the majority of surgeons preferred a straight electrode array when attempting to preserve residual acoustic hearing.^57^ The rationale behind this preference stems from the physical properties and trajectory of insertion of straight electrodes, which are designed to atraumatically track along the outer wall of the scala tympani during insertion, thereby minimizing contact with the osseous spiral lamina and basilar membrane.^35^

Earlier studies examining the relationship between electrode array design and hearing preservation generally supported the preference for straight electrodes in patients with residual acoustic hearing. In a cohort of 225 implants, Wanna et al and Thompson et al described higher hearing preservation rates among recipients of straight electrodes.^43^ Multiple studies have since concluded that straight arrays are more commonly associated with short‐term preservation of low‐frequency hearing, likely due to their less traumatic insertion profile.^50^, ^58^, ^59^ However, these short‐term advantages may diminish overtime, with long‐term outcomes showing more modest benefit. Notably, many of these earlier comparisons involved precurved electrodes that were inserted using a rigid stylet, which differ in design and insertion mechanics from newer‐generation sheath‐based insertion arrays like the CI532/632.^52^, ^60^ We hypothesize that precurved arrays, with a sheath‐like delivery system, achieve curvature without contacting the lateral wall due to their modiolar‐hugging trajectory, which reduces the mechanical disruption of cochlear fluids. Conversely, straight electrodes necessarily exert pressure on the lateral wall to initiate curvature. These mechanics may allow for improved hearing preservation after precurved electrode insertion, particularly in the short‐term.

In our study, we observed that ears implanted with a slim precurved electrode exhibited a significantly lower average LFPTA shift compared to those implanted with a straight electrode at 1‐ and 6‐months, indicating greater preservation of low‐frequency hearing in the short‐term. The precurved electrode advantage diminished in long‐term follow‐up, with precurved and straight arrays demonstrating similar rates of hearing preservation. These results contribute to a growing body of evidence suggesting that modern precurved designs—such as the CI532/632 electrode arrays—may mitigate some of the intracochlear trauma historically associated with older precurved electrodes and provide hearing preservation outcomes that are comparable to straight arrays. Sharma et al analyzed hearing preservation between precurved and straight electrode groups in a group of 116 patients, and demonstrated no significant difference in LFPTA shift at 6 months or 12 months postoperatively after controlling for age, sex, and time of follow‐up.^37^ Woodson et al assess both absolute LFPTA shift and “successful” hearing preservation—defined as postoperative LFPTA < 80 dB HL—and reported similar results between slim precurved and straight electrode recipients.^61^ Similarly, Zhan et al also found no significant differences in functional hearing preservation rates between precurved and straight arrays at activation or at 1 year postoperatively.^47^ Taken together, these studies suggest that the hearing preservation gap between precurved and straight electrodes has narrowed.

### Speech Perception Outcomes

While CNC and AzBio scores were numerically higher in the precurved group at nearly all time points, these differences were not statistically significant at any measured interval. This trend aligns with findings from several recent studies. Sharma et al reported superior CNC scores in the precurved group compared to the straight group and a nonsignificant trend toward better AzBio performance in aggregate analysis.^37^ Notably, their sensitivity analysis revealed that the AzBio advantage reached statistical significance at the 12‐month timepoint. Vohra et al and Pennington‐FitzGerald et al investigated precurved and straight electrodes across several manufacturers and reported higher CNC scores in patients with precurved electrodes.^38^, ^39^ Similarly, other authors have reported comparable or higher speech perception outcomes for precurved electrodes compared to straight electrodes, both in short‐ and long‐term follow up.^36^, ^41^, ^49^

Several other studies, however, have demonstrated comparable speech perception outcomes in recipients with precurved and straight electrodes, in which no statistically significant differences were observed between groups at any postoperative timepoint. Briggs et al found no significant effect of electrode array position for any speech perception results, nor any relationship between angle (depth) of electrode array insertion and speech perception outcomes.^32^ Similarly, Moran et al reported equivalent outcomes following implantation with straight and precurved electrodes in ears with functional preserved low‐frequency hearing.^42^ MacPhail et al demonstrated comparable improvements in AzBio scores in quiet between precurved and straight recipients when matched for preoperative performance.^46^ Fabie et al found no significant differences in postoperative CNC, AzBio in quiet, or AzBio in noise based on electrode type after adjusting for preoperative hearing and speech recognition.^48^

### Other Factors Influencing Cochlear Implant Outcomes

Multivariate analysis in our study revealed that younger age at implantation was significantly associated with better CNC and AzBio scores at 1‐, 3‐, and 6‐month postactivation. This is consistent with a robust body of literature demonstrating that earlier implantation is linked to superior speech perception outcomes.^13^, ^14^ Similarly, hearing preservation was more frequently achieved in younger patients within our cohort, a trend that has also been reported across multiple other studies.^50^, ^62^, ^63^

## Limitations

There are several important limitations. Audiologic follow‐up was limited to 1 year; ideally, longer‐term data would allow for a more comprehensive assessment of outcomes and potential differences between precurved and straight electrodes overtime. Nevertheless, short‐term differences may yield noticeable benefits for CI recipients. For example, we observed significantly better hearing preservation in the precurved group at 1‐ and 6‐month postoperatively, though this benefit diminished with longer follow‐up. Notably, patients who experienced a profound loss of hearing postoperatively at any timepoint did not undergo repeat threshold testing at later intervals, which may explain the increasing percentages of hearing preservation over time within our cohort.

Additionally, this study evaluated devices from a single manufacturer to reduce variability across electrode designs, but this limits the generalizability of our findings to other manufacturers. All procedures were performed at a single institution by 3 implanting surgeons and electrode type was chosen based upon surgeon preference; we could not randomize due to the retrospective nature of the study. We were also unable to directly assess intracochlear electrode position, insertion depth, or scalar translocation. Our clinic does not routinely collect data on whether patients are active EAS users and therefore we could not control for EAS versus CI‐alone testing, which may introduce potential bias into speech recognition comparison findings. Finally, AzBio scores in noise were not routinely collected and therefore not included in our analysis, though this is an important functional outcome for real‐world listening.

## Conclusion

Our study presents compelling evidence for superior short‐term hearing preservation in cochlear implantation with precurved electrode arrays as compared to straight electrode arrays, which validates the use of newer design models and surgical techniques. In our cohort, speech recognition outcomes globally improved after cochlear implantation, but these gains were not influenced by electrode design. Taken together, these findings suggest that precurved electrodes may deliver early benefits with respect to hearing preservation, while performing similarly to straight arrays over time.

## Author Contributions

**Lily V. Den Hartog**, conception, data acquisition and analysis, initial manuscript preparation; **Andrew W. Liu**, conception, data analysis, initial manuscript preparation; **Divya A. Chari**, conception, interpretation of data, manuscript revisions and final approval.

## Disclosures

### Competing interests

None.

### Funding source

None.
